# Supplementary material for: Comparison of laboratory-based and non-laboratory-based WHO and GLOBORISK CVD risk scores: A cross-sectional analysis of the APCAPS cohort
Source: PLoS One. 2026 Feb 6;21(2):e0342471. doi: 10.1371/journal.pone.0342471 (PMC12880651; doi:10.1371/journal.pone.0342471)
Supplement: Attachment — Submitted filename: PONE-D-25-38253_hutton.pdf [file pone.0342471.s010.pdf]

PLOS ONE

COMPARISON OF LABORATORY-BASED AND NON-LABORATORY-BASED WHO AND GLOBORISK CVD RISK SCORES: A CROSS-SECTIONAL ANALYSIS OF THE APCAPS COHORT

--Manuscript Draft--

**Manuscript Number:**	PONE-D-25-38253
**Article Type:**	Research Article
**Full Title:**	COMPARISON OF LABORATORY-BASED AND NON-LABORATORY-BASED WHO AND GLOBORISK CVD RISK SCORES: A CROSS-SECTIONAL ANALYSIS OF THE APCAPS COHORT
**Short Title:**	LABORATORY-BASED AND NON-LABORATORY-BASED WHO AND GLOBORISK CVD RISK SCORES FOR APCAPS
**Corresponding Author:**	Hemant Mahajan, MD<br>National Institute of Nutrition<br>Patna, Bihar INDIA
**Keywords:**	Cardiovascular diseases; risk prediction models; non-laboratory-based algorithms; WHO-CVD risk score; GLOBORISK; India
**Abstract:**	Background and Aim: Cardiovascular diseases (CVDs) represent a growing public-health challenge in India, where nearly one in four deaths is CVD-related. Accurate risk stratification underpins targeted prevention, yet laboratory-dependent tools are often impractical in resource-limited settings. The World Health Organization (WHO) and GLOBORISK initiatives both offer non-laboratory-based 10-year CVD risk algorithms alongside their laboratory-based counterparts. We aimed to compare laboratory- and non-laboratory-based WHO and GLOBORISK CVD risk scores, assess their concordance, and examine relationships with sub-clinical atherosclerosis in a rural Indian cohort.<br>Materials and Methods: We conducted a cross-sectional analysis of 2,465 adults (1,184 men, 1,281 women) aged 40-74 years from the third wave (2010-12) of the Andhra Pradesh Children and Parents Study (APCAPS). Participants with prior CVD were excluded. Ten-year CVD risk was calculated using sex-specific WHO (South Asia) and India-calibrated GLOBORISK models, both laboratory-based (age, sex, smoking, systolic blood pressure, diabetes, total cholesterol) and non-laboratory-based (age, sex, smoking, systolic blood pressure, BMI) algorithms. Categorical agreement was quantified via percentage agreement and quadratic weighted kappa (κ); continuous agreement by Bland-Altman analysis. We also evaluated linear associations between each risk score (categorical and continuous) and three sub-clinical atherosclerosis markers: carotid intima-media thickness (CIMT), pulse-wave velocity (PWV), and augmentation index (AIx), through sex-stratified linear regression, adjusting for multiple testing (p<0.01).<br>Results: Median WHO risk was 6.0% (IQR 4% - 9%) in men and 3.0% (2% - 4%) in women for both lab and non-lab models; median GLOBORISK risk was 12.0% (9% - 16%) vs. 15.0% (10% - 16%) in men and 5.0% (3% -9%) vs. 5.0% (3% - 9%) in women. Categorical agreement was substantial to almost perfect: WHO κ=0.82 (overall), GLOBORISK κ=0.72. Bland-Altman analyses demonstrated mean differences <1% between lab- and non-lab-based scores, though non-lab models underestimated risk by 4.2% in diabetics and 1.2% in participants with total cholesterol ≥200 mg/dL. Both risk scores showed positive, dose-response relationships with CIMT, PWV, and AIx (p-trend<0.001), with each SD increase in score associated with clinically meaningful increases in all three markers.<br>Conclusion: Non-laboratory-based WHO and GLOBORISK CVD risk scores exhibit high overall agreement with laboratory-based models and correlate strongly with subclinical atherosclerosis in rural India. However, modest underestimation in high-risk subgroups (diabetics, hypercholesterolemia) warrants cautious interpretation. These findings support the feasibility of non-lab risk assessment in resource-constrained settings, while underscoring the need for prospective validation against hard cardiovascular outcomes prior to large-scale implementation.
**Order of Authors:**	Hemant Mahajan, MD, DrPH

Powered by Editorial Manager® and ProduXion Manager® from Aries Systems Corporation

	Poppy Alice Carson Mallinson, PhD
	Judith Lieber, PhD
	Santhi Bhogadi, MCA
	Santosh Kumar Banjara, MD
	Anoop Shah, MD, PhD
	Vipin Gupta, PhD
	Gagandeep Kaur Walia, PhD
	Bharati Kulkarni, PhD
	Sanjay Kinra, MD, PhD
**Additional Information:**	
**Question**	**Response**
**Financial Disclosure**<br><br>Enter a financial disclosure statement that describes the sources of funding for the work included in this submission. Review the submission guidelines for detailed requirements. View published research articles from *PLOS ONE* for specific examples.<br><br>This statement is required for submission and **will appear in the published article** if the submission is accepted. Please make sure it is accurate.<br><br>**Funded studies**<br>Enter a statement with the following details:<br>• Initials of the authors who received each award<br>• Grant numbers awarded to each author<br>• The full name of each funder<br>• URL of each funder website<br>• Did the sponsors or funders play any role in the study design, data collection and analysis, decision to publish, or preparation of the manuscript?<br><br>Did you receive funding for this work?	The author(s) received no specific funding for this work.
**Competing Interests**<br><br>Use the instructions below to enter a competing interest statement for this submission. On behalf of all authors, disclose any competing interests that	NO authors have competing interests

could be perceived to bias this work–acknowledging all financial support and any other relevant financial or non-financial competing interests.<br><br>This statement is required for submission and **will appear in the published article** if the submission is accepted. Please make sure it is accurate and that any funding sources listed in your Funding Information later in the submission form are also declared in your Financial Disclosure statement.<br><br>View published research articles from *PLOS ONE* for specific examples.<br><br>**NO authors have competing interests**<br><br>Enter: *The authors have declared that no competing interests exist.*<br><br>**Authors with competing interests**<br><br>Enter competing interest details beginning with this statement:<br><br>*I have read the journal's policy and the authors of this manuscript have the following competing interests: [insert competing interests here]*<br><br>* typeset	
**Ethics Statement**<br><br>Enter an ethics statement for this submission. This statement is required if the study involved:<br><br>• Human participants<br>• Human specimens or tissue<br>• Vertebrate animals or cephalopods<br>• Vertebrate embryos or tissues<br>• Field research<br><br>Write "N/A" if the submission does not require an ethics statement.	Ethical approval was granted by the London School of Hygiene & Tropical Medicine (London, UK) [LSHTM Ethics Ref: 21771], the ICMR-National Institute of Nutrition (Hyderabad, India) [NIN Protocol Number: 04/01/2020], and the Indian Institute of Public Health (Hyderabad, India) [IIPHH/TRCIEC/189/2018]. Permissions from village heads and governing committees were obtained verbally, and informed written consent (or a witnessed thumbprint for illiterate participants) was collected from all participants prior to their enrolment in the study.

Powered by Editorial Manager® and ProduXion Manager® from Aries Systems Corporation

General guidance is provided below. Consult the submission guidelines for detailed instructions. **Make sure that all information entered here is included in the Methods section of the manuscript.**<br><br>**Format for specific study types**<br><br>**Human Subject Research (involving human participants and/or tissue)**<br>• Give the name of the institutional review board or ethics committee that approved the study<br>• Include the approval number and/or a statement indicating approval of this research<br>• Indicate the form of consent obtained (written/oral) or the reason that consent was not obtained (e.g. the data were analyzed anonymously)<br><br>**Animal Research (involving vertebrate animals, embryos or tissues)**<br>• Provide the name of the Institutional Animal Care and Use Committee (IACUC) or other relevant ethics board that reviewed the study protocol, and indicate whether they approved this research or granted a formal waiver of ethical approval<br>• Include an approval number if one was obtained<br>• If the study involved *non-human primates*, add *additional details* about animal welfare and steps taken to ameliorate suffering<br>• If anesthesia, euthanasia, or any kind of animal sacrifice is part of the study, include briefly which substances and/or methods were applied<br><br>**Field Research**<br><br>Include the following details if this study involves the collection of plant, animal, or other materials from a natural setting:<br>• Field permit number<br>• Name of the institution or relevant body that granted permission	
**Data Availability**<br><br>Authors are required to make all data	Yes - all data are fully available without restriction

Powered by Editorial Manager® and ProduXion Manager® from Aries Systems Corporation

underlying the findings described fully available, without restriction, and from the time of publication. PLOS allows rare exceptions to address legal and ethical concerns. See the PLOS Data Policy and FAQ for detailed information.<br><br>A Data Availability Statement describing where the data can be found is required at submission. Your answers to this question constitute the Data Availability Statement and **will be published in the article**, if accepted.<br><br>**Important:** Stating 'data available on request from the author' is not sufficient. If your data are only available upon request, select 'No' for the first question and explain your exceptional situation in the text box.<br><br>Do the authors confirm that all data underlying the findings described in their manuscript are fully available without restriction?	
**Describe where the data may be found in full sentences. If you are copying our sample text, replace any instances of XXX with the appropriate details.**<br><br>• If the data are **held or will be held in a public repository**, include URLs, accession numbers or DOIs. If this information will only be available after acceptance, indicate this by ticking the box below. For example: *All XXX files are available from the XXX database (accession number(s) XXX, XXX.).*<br>• If the data are all contained **within the manuscript and/or Supporting Information files**, enter the following: *All relevant data are within the manuscript and its Supporting Information files.*<br>• If neither of these applies but you are able to provide **details of access elsewhere**, with or without limitations, please do so. For example:<br><br>*Data cannot be shared publicly because of [XXX]. Data are available from the*	Data cannot be shared publicly because of Privacy policy of the Indian Council of Medical Research, India. Data are available from the ICMR-NIN Institutional Data Access / Ethics Committee (contact via email) for researchers who meet the criteria for access to confidential data.

Powered by Editorial Manager® and ProduXion Manager® from Aries Systems Corporation

XXX Institutional Data Access / Ethics Committee (contact via XXX) for researchers who meet the criteria for access to confidential data.<br><br>*The data underlying the results presented in the study are available from (include the name of the third party and contact information or URL).*<br>• This text is appropriate if the data are owned by a third party and authors do not have permission to share the data.<br><br>* typeset	
Additional data availability information:	

Powered by Editorial Manager® and ProduXion Manager® from Aries Systems Corporation

Manuscript

Click here to access/download;Manuscript;CVD_WHO_GLOBORISK_Manu

COMPARISON OF LABORATORY-BASED AND NON-LABORATORY-BASED WHO AND GLOBORISK CVD RISK SCORES: A CROSS-SECTIONAL ANALYSIS OF THE APCAPS COHORT

Hemant Mahajan[1*], Poppy Alice Carson Mallinson[2*], Judith Lieber[2], Santhi Bhogadi[1], Santosh Kumar Banjara[1], Anoop Shah[2], Vipin Gupta[3], Gagandeep Kaur Walia[4], Bharati Kulkarni[1], Sanjay Kinra[2]

Authors' Affiliation:

[1]Indian Council of Medical Research - National Institute of Nutrition, Hyderabad, India

[2]London School of Hygiene & Tropical Medicine, London, UK

[3]University of Delhi, Delhi, India

[4]Public Health Foundation of India, Delhi, India

[*]**Corresponding Author**

Dr. Hemant Mahajan; hemant.mahajan.84@gmail.com+91-9666954994

Dr. Poppy Alice Carson Mallinson; Poppy.Mallinson1@lshtm.ac.uk +44 7875866640

Abstract

Background and Aim: Cardiovascular diseases (CVDs) represent a growing public-health challenge in India, where nearly one in four deaths is CVD-related. Accurate risk stratification underpins targeted prevention, yet laboratory-dependent tools are often impractical in resource-limited settings. The World Health Organization (WHO) and GLOBORISK initiatives both offer non-laboratory-based 10-year CVD risk algorithms alongside their laboratory-based counterparts. We aimed to compare laboratory- and non-laboratory-based WHO and GLOBORISK CVD risk scores, assess their concordance, and examine relationships with sub-clinical atherosclerosis in a rural Indian cohort.

Materials and Methods: We conducted a cross-sectional analysis of 2,465 adults (1,184 men, 1,281 women) aged 40–74 years from the third wave (2010–12) of the Andhra Pradesh Children and Parents Study (APCAPS). Participants with prior CVD were excluded. Ten-year CVD risk was calculated using sex-specific WHO (South Asia) and India-calibrated GLOBORISK models, both laboratory-based (age, sex, smoking, systolic blood pressure, diabetes, total cholesterol) and non-laboratory-based (age, sex, smoking, systolic blood pressure, BMI) algorithms. Categorical agreement was quantified via percentage agreement and quadratic weighted kappa (κ); continuous agreement by Bland–Altman analysis. We also evaluated linear associations between each risk score (categorical and continuous) and three sub-clinical atherosclerosis markers: carotid intima-media thickness (CIMT), pulse-wave velocity (PWV), and augmentation index (AIx), through sex-stratified linear regression, adjusting for multiple testing ($p<0.01$).

Results: Median WHO risk was 6.0% (IQR 4% - 9%) in men and 3.0% (2% - 4%) in women for both lab and non-lab models; median GLOBORISK risk was 12.0% (9% - 16%) vs. 15.0% (10% - 16%) in men and 5.0% (3% -9%) vs. 5.0% (3% - 9%) in women. Categorical agreement was substantial to almost perfect: WHO κ=0.82 (overall), GLOBORISK κ=0.72. Bland-Altman analyses demonstrated mean differences <1% between lab- and non-lab-based scores, though non-lab models underestimated risk by 4.2% in diabetics and 1.2% in participants with total cholesterol ≥200 mg/dL. Both risk scores showed positive, dose–response relationships with CIMT, PWV, and AIx (p-trend<0.001), with each SD increase in score associated with clinically meaningful increases in all three markers.

Conclusion: Non-laboratory-based WHO and GLOBORISK CVD risk scores exhibit high overall agreement with laboratory-based models and correlate strongly with subclinical atherosclerosis in rural India. However, modest underestimation in high-risk subgroups (diabetics, hypercholesterolemia) warrants cautious interpretation. These findings support the feasibility of non-lab risk assessment in resource-constrained settings, while underscoring the need for prospective validation against hard cardiovascular outcomes prior to large-scale implementation.

Keywords: Cardiovascular diseases, Risk prediction models, non-laboratory-based algorithms, WHO-CVD risk score, GLOBORISK, India

INTRODUCTION

Cardiovascular diseases (CVD) are the leading global cause of death, with a growing burden in developing countries like India. Nearly a quarter of all deaths in India are attributed to CVD, with an age-standardized death rate of 272 per 100,000 population, higher than the global average of 235 per 100,000 [1]. CVD prevalence in India has increased fivefold over three decades, accounting for 26.6% of total deaths and 13.6% of disability-adjusted life years (DALYs) in 2017, a significant rise from 1990 [2, 3]. This escalating burden poses significant economic and healthcare challenges, highlighting the need for effective primary prevention strategies.

CVD predicting risk scores such as the Framingham Risk Score (FRS) , INTERHEART Modifiable Risk Score (IHMRS) [4], WHO-CVD risk charts, SCORE, GLOBORISK, and the Revised Pooled Cohort Equation (RPCE), among others [2, 5-10] assess CVD risk by considering the synergistic effects of risk factors like hypertension, diabetes, dyslipidaemia, obesity, and smoking. These cost-effective tools enable personalized risk mitigation through lifestyle modifications (such as increased physical activity, heart-healthy diets, and smoking cessation) and medications (lipid-lowering, blood pressure-lowering, and anti-thrombotic) to reduce CVD-related morbidity and mortality. However, most of these risk scores are based on data from high-income countries and may not accurately estimate CVD risk for the Indian population due to different risk factor profiles and CVD event rates [2].

Given the lack of an indigenous CVD prediction score in India, GLOBORISK calibrated to Indian data [10] or WHO-CVD risk scores developed for South Asia are commonly used [5]. Both provide non-laboratory-based algorithms (in addition to lab-based algorithm) that rely on easily obtainable variables such as age, sex, smoking status, systolic blood pressure (SBP), and body mass index (BMI), making them suitable for use in resource-limited settings without laboratory infrastructure. These non-laboratory-based algorithms can be integrated into India's Non-Communicable Disease (NCD) solution applications—mobile and tablet-based platforms developed for NCD prevention, screening, and management [11, 12]. For example, as part of the Comprehensive Primary Health Care initiative in Telangana, Accredited Social Health Activists (ASHAs) use the state government's 'ASHA Disease Profile' app to capture data on risk factors and symptoms of communicable and non-communicable diseases across the population [13].

Despite the advantages of non-laboratory-based CVD prediction algorithms, their performance relative to laboratory-based models have not been extensively evaluated in Indian populations. Some studies [14-16] have assessed their predictive ability, but limitations such as small sample sizes, lack of sex-stratified analyses, absence of validation using clinical or subclinical CVD markers, reliance on point-of-care devices for blood sugar measurement, and restriction of analysis to individuals aged 30-69 years (despite the WHO-CVD risk score being designed for individuals aged 40-74 years) have constrained their generalizability. Notably, Phillip et al. [17] using data from the PURE study, developed region-specific non-laboratory-based IHMRS models, including one for South Asia. Their findings demonstrated that non-laboratory-based risk scores can perform comparably to laboratory-based models. However, the IHMRS was specifically designed for myocardial infarction (MI) using a case-control study and requires multiple variables-including age, sex, diabetes, hypertension, smoking history, second-hand smoke exposure, physical activity, depression, general stress, waist-to-hip ratio, diet, and meat intake making it more complex than WHO-CVD risk scores or GLOBORISK.

In this study, we aimed to estimate predicted CVD risk in the Andhra Pradesh Children and Parents Study (APCAPS) cohort using the GLOBORISK CVD score recalibrated for India [10] and updated WHO-CVD risk prediction tools [5]. We also sought to assess the concordance between laboratory-based and non-laboratory-based scores, offering insights into their utility in resource-constrained Indian settings.

Commented [KH1]: What about their relationship with subclinical atherosclerosis?

METHODS

Study Population

The APCAPS is based in 29 rural villages and towns located approximately 50 km from Hyderabad. Initially, APCAPS was established as a long-term follow-up study of the offspring from a pregnancy nutrition trial conducted between 1987 and 1990. Over time, the study expanded to include family members of the original trial participants. For the present analysis, we utilized cross-sectional data from the third wave of data collection (2010–2012), during which 6,944 of the 10,213 (68%) invited family members participated in clinical examinations [18]. Data for the third wave of APCAPS were accessed for the current analysis on 01/01/2025 (PI: Prof. Sanjay Kinra). The study adhered to the ethical principles outlined in the Declaration of Helsinki. Ethical approval was granted by the London School of Hygiene & Tropical Medicine (London, UK) [LSHTM Ethics Ref: 21771], the ICMR-National Institute of Nutrition (Hyderabad, India) [NIN Protocol Number: 04/01/2020], and the Indian Institute of Public Health (Hyderabad, India) [IIPHH/TRCIEC/189/2018]. Permissions from village heads and governing committees were obtained verbally, and informed written consent (or a witnessed thumbprint for illiterate participants) was collected from all participants prior to their enrolment in the study.

Commented [KH2]: Is this still an on-going study. If no, then it should be "was"

Clinical Assessment

As previously published [18, 19], trained interviewers collected data on tobacco and alcohol use at village clinics using standard questions from India's Third National Family Health Survey. Socioeconomic status was assessed with 14 selected questions from the Standard of Living Index (SLI), where higher scores indicated higher socioeconomic status. Weight and height were measured twice using standard protocols, with discrepancies prompting a third reading. BMI was calculated as weight (kg) divided by height (m^2). Blood pressure was measured in the right arm using an Omron M5-I oscillometric device after a 5-minute rest. Three readings were taken one minute apart, with the mean of the last two used for analysis. Hypertension was defined as a prior clinical diagnosis and/or SBP ≥140 mmHg or diastolic blood pressure (DBP) ≥90 mmHg.

Venous blood samples were collected after an 8-hour fast, and glucose was measured using the GOD-PAP method on the same day. Lipids (total cholesterol, triglycerides, high density lipoprotein-cholesterol (HDL-C)) were quantified using enzymatic assays, with quality maintained through internal controls and participation in external quality programs. Assays had intra- and inter-assay variation coefficients below 3% and 5%, respectively [18]. Diabetes was defined as a prior clinical diagnosis and/or fasting glucose ≥126 mg/dL. The reliability of clinical measurements was ensured by strict protocols, regular calibration, and monitoring for consistency. Reproducibility, assessed with repeat measurements in a random 5% subsample, showed intraclass correlation coefficients of >0.98 for anthropometry, >0.85 for vascular measures, and >0.94 for biochemical assays [18].

Commented [KH3]: Was LDL-C derived?

CVD-risk prediction scores: WHO and GLOBORISK

The 10-year CVD risk was calculated using both laboratory-based and non-laboratory-based charts for WHO CVD-risk prediction (South Asia) and GLOBORISK (India) for males and females aged 40 to 74 years, without a self-reported history of CVD (heart disease or coronary bypass surgery or stroke) [5, 10]. For both the CVD risk prediction scores (WHO as well as GLOBORISK) the sex-specific laboratory-based model included the variables age, smoking, diabetes, SBP and total cholesterol, while the sex-specific non-laboratory-based model included age, smoking, SBP,

Commented [KH4]: Did you consider peripheral artery disease?

and BMI [5]. Analysis was restricted to participants age 40 to 74 years, and complete data for age, SBP, BMI, fasting glucose, and total cholesterol. The endpoints used to develop both the CVD risk prediction scores were: fatal or non-fatal myocardial infarction or coronary heart disease, and fatal or non-fatal stroke over a 10-year period (Figure 1.0) [5]. For both the CVD risk prediction scores, an individual is grouped into the '*high'* cardiovascular risk with a 10-year risk of $\geq$20%. '*Low'* risk was defined as cardiovascular risk with a 10-year risk of $<$10%. All other risk values were categorized as the '*intermediate'* [5, 10, 20].

The validity of WHO-CVD and GLOBORISK risk prediction scores was assessed separately for males and females using measures of subclinical atherosclerosis - Carotid Intima-Media Thickness (CIMT), Pulse Wave Velocity (PWV), and Augmentation Index (AIx), which are considered precursors of CVD. Standardized clinical procedures for measuring CIMT, PWV, and AIx have been documented elsewhere [21]

Statistical Analyses

Categorical variables were expressed as numbers (percentages), and continuous variables were presented as means and standard deviations (SDs). Linear regression analysis was conducted separately for males and females to explore the association between WHO and GLOBORISK CVD risk scores and measures of subclinical atherosclerosis. CVD risk scores were considered both as categorical variables (classified into low, intermediate, and high-risk categories) and as continuous variables to assess their relationship with subclinical atherosclerosis markers. To examine trends across CVD risk categories, median values were assigned to each category and treated as a continuous variable. Additionally, the change in subclinical atherosclerosis markers per SD increase in CVD scores was estimated separately for males and females. All p-values were two-tailed and a p-value less than 0.01 was considered significant to account for multiple testing.

Categorical CVD risk agreement was assessed using percent agreement and quadratic weighted kappa statistics [22], while Bland-Altman plots evaluated the agreement between continuous CVD risk scores from laboratory-based and non-laboratory-based models [23]. The Kappa values were classified as poor ($\leq$ 0), slight (0.01-0.20), fair (0.21-0.40), moderate (0.41-0.60), substantial (0.61-0.80), and almost perfect (0.81-1.0) [24]. For Bland-Altman plots, the difference in risk (laboratory-based minus non-laboratory-based) and the mean risk scores were calculated. These plots were stratified by sex, and mean differences with 95% confidence intervals (CIs) were computed using paired t-tests. The plots show the relationship between the difference in scores (vertical axis) and their mean (horizontal axis). Limits of agreement, defined as the mean difference $\pm$ 1.96 SD of score differences, were also presented [23]. The quadratic weighted Kappa statistics and Bland-Altman analysis were performed overall, as well as separately for males, females, and participants with specific risk factors, including smoking, diabetes, hypertension, BMI $\geq$ 25 kg/m^2, and total cholesterol $\geq$ 200 mg/dL.

Commented [KH5]: Was this done as part of the earlier study in 2010-2012 or this was done by the current authors?

RESULTS

CVD-Risk Score Analysis

A total of 1,184 males and 1,281 females were analyzed for the WHO and GLOBORISK CVD-risk score. The mean (SD) age was 53.47 years (6.73) for males and 47.22 years (5.47) for females. Among males, the median (IQR) lab- and non-lab-based WHO CVD-risk scores were both 6.0% (4.0, 9.0), with 22 to 25% of males having a predicted risk ≥10%. For females, the median (IQR) lab- and non-lab-based WHO CVD-risk score was 3.0% (2.0, 4.0), with 3% having a predicted risk ≥10%. Whereas, for males' median (IQR) lab- and non-lab-based GLOBORISK CVD-risk scores were 12.0 (9.0, 16.0) and 15.0 (10.0, 16.0), respectively. Approximately 70% and 80% males were having lab- and non-lab-based GLOBORISK CVD-risk scores ≥10%. For females, the median (IQR) lab- and non-lab-based GLOBORISK CVD-risk score was 5.0 (3.0, 9.0) with approximately 21% having a predicted lab-based risk and approximately 24% having a predicted non-lab-based risk ≥10% (Table 1).

The mean (SD) values for subclinical atherosclerosis markers were: CIMT was 1.001 mm (0.215), PWV was 8.02 m/s (1.34), and AIx was 29.44% (8.38) in males. Among females, CIMT was 0.985 mm (0.182), PWV was 7.64 m/s (1.24), and AIx was 30.40% (9.11) (Table 1). There was a linear and positive association between CVD-prediction scores (WHO-CVD and GLOBORISK CVD risk scores) and the measures of subclinical atherosclerosis. The levels of CIMT, PWV, and AIx were increasing with the increase in the severity levels of CVD-prediction scores (low, intermediate and high CVD-risk for WHO as well as GLOBORISK, $p_{linear\text{-}trend} <0.05$). One SD increase in CVD scores were significantly associated with increase in levels of CIMT, PWV, and AIx for both males and females across WHO as well GLOBORISK CVD risk scores (Table 2).

Categorical agreement and Kappa Coefficients

For the WHO CVD risk scores, the ~~percent~~ percentage agreement between risk categories was 98.6% with a quadratic weighted kappa (95% CI) of 0.82 (0.78, 0.85). The quadratic weighted kappa (95% CI) among males and females were 0.81 (0.77, 0.85) and 0.73 (0.62, 0.84), respectively. When analysed across different risk factors, quadratic weighted kappa was substantial to almost perfect except among participants diagnosed with diabetes. The percentage agreement between risk categories was 91.0% and the quadratic weighted kappa (95% CI) was 0.53 (0.42, 0.64) among participants diagnosed with diabetes (Table 3).

Formatted: Highlight

For the GLOBORISK CVD risk scores, the ~~percent~~ percentage agreement between risk categories was 94.0% with a Quadratic Weighted Kappa (95% CI) of 0.72 (0.69, 0.74). The quadratic weighted kappa (95% CI) among males and females were 0.63 (0.59, 0.67) and 0.63 (0.58, 0.68), respectively. When analysed across different risk factors, Quadratic Weighted Kappa was moderate to substantial except among participants diagnosed with diabetes and among smokers. The Quadratic Weighted Kappa (95% CI) was 0.42 (0.35, 0.49) among participants diagnosed with diabetes and 0.48 (0.43 to 0.52) among smokers (Table 3).

Bland–Altman Plots: Mean Differences and Limits of Agreement

For the WHO CVD risk scores, the mean difference between lab-based and non-lab-based scores was 0.10 (0.03, 0.17). The limits of agreement (lower and upper) were -31.9 and 3.39, respectively. Among males and females, the

mean difference between lab-based and non-lab-based scores were 0.31% (95% CI: 0.19, 0.43) and -0.09% (95% CI: -0.15, -0.03), respectively. The limits of agreement (lower and upper) were -3.87% to 4.49% for males and -2.19% to 2.01% for females (Table 4, Supplementary figures 1A, 1B, 1C). When analysed across different risk factors, the mean difference between lab-based and non-lab-based scores was less than 1% except participants diagnosed with diabetes [mean difference (95% CI): 4.17% (3.76%, 4.58%)] and participants with total cholesterol ≥200 mg/dL [mean difference (95% CI): 1.16% (0.98%, 1.34%)].

For the GLOBORISK CVD risk scores, the mean difference between lab-based and non-lab-based scores was -0.53 (-0.70, -0.36). The limits of agreement (lower and upper) were -9.15 and 8.09, respectively. Among males and females, the mean difference between lab-based and non-lab-based scores were -0.66% (95% CI: -0.95%, -0.37%) and -0.41% (95% CI: -0.61%, -0.21%), respectively. The limits of agreement (lower and upper) were -10.62% to 9.30% for males and -7.56% to 6.74% for females (Table 4, Supplementary figures 2A, 2B, 2C). When analysed across different risk factors, the mean difference between lab-based and non-lab-based scores was less than 1% except for smokers [mean difference (95% CI): -1.15% (-1.48%, -0.82%)], participants diagnosed with diabetes [mean difference (95% CI): 10.44% (9.38%, 11.50%)] and participants with total cholesterol ≥200 mg/dL [mean difference (95% CI): 2.85% (2.38%, 3.32%)].

DISCUSSION

The cross-sectional analysis from the APCAPS third follow-up estimated the 10-year CVD risk using both laboratory-based and non-laboratory-based algorithms among participants aged 40 to 74 years residing in southern India without a prior CVD diagnosis. The analysis revealed a positive linear association between WHO and GLOBORISK CVD risk scores and markers of subclinical atherosclerosis. Overall, there was substantial to almost perfect agreement (72% - 82%) between lab-based and non-lab-based risk scores for both models, with consistent agreement observed among men and women (63% - 81%). Bland-Altman analysis showed that the mean difference between the two methods was less than 1% for both WHO and GLOBORISK CVD risk assessments, indicating strong concordance. However, the level of agreement varied across specific risk factor subgroups such as participants diagnosed with diabetes mellitus or participants with total cholesterol levels ≥200 mg/dL.

Commented [KH6]: Aren't smokers also having varied level of agreement?

Our finding of substantial to almost perfect agreement between lab-based and non-lab-based risk scores aligns with previous studies conducted in India [14-16], as well as research by Bendera et al. in Eastern Sub-Saharan Africa [25], Dehghan et al. and Rezaei et al. in Iran [26, 27] and Chhezom et al. in Bhutan [28], validating the effectiveness of non-laboratory-based methods in resource-limited settings. Furthermore, Phillip et al. [17] using data from the PURE study spanning seven distinct global regions, demonstrated that non-laboratory-based tools exhibit predictive accuracy comparable to laboratory-based methods. Collectively, these findings reinforce the reliability of simplified, non-lab-based models for CVD risk assessment across diverse populations. Consistent with previous research, our results support the broader applicability of these models, particularly in settings with limited laboratory resources or access.

Commented [KH7]: There is also a study among the West African population by Hutton et al comparing lab vs non-lab CVD risk scores that you can cite

Commented [KH8]: This broader applicability is limited to non-diabetics

Although the agreement between lab-based and non-lab-based CVD risk scores was substantial, Bland–Altman analysis revealed systematic bias, with non-laboratory-based methods consistently underestimating or overestimating risk compared to laboratory-based models. For WHO-CVD, the mean difference (95% CI) was 0.10 (0.03, 0.17), while for GLOBORISK, it was -0.53 (-0.70, -0.36). Despite the mean difference being less than 1%, the line of equality fell outside the confidence interval in all cases, indicating a persistent bias. Additionally, the variability of differences between the two methods increased with higher risk estimates (observed for both WHO-CVD and GLOBORISK CVD scores, Supplementary figures 1A, 1B, 1C, 2A, 2B, 2C). To further assess this trend, we plotted the proportional difference [(Lab-based Score – Non-lab-based Score)/Mean×100] against the mean of the two measurements. However, systematic bias persisted across all levels of risk estimation, suggesting that non-laboratory-based models tend to underestimate CVD risk, particularly in individuals with intermediate-to-high risk profiles. The extent of bias was notably greater in participants with total cholesterol ≥200 mg/dL (1% - 3%) and in those with diabetes (5% - 11%). Kappa analysis further indicated only moderate concordance (< 50%) between lab-based and non-lab-based models in diabetic individuals, consistent with previous studies [5, 10, 16]. Bland–Altman analysis confirmed that non-lab-based scores systematically underestimated CVD risk in those with diabetes, especially among individuals with higher overall risk levels (Supplementary figures 3A, 3B). This underestimation raises concerns that high-risk individuals classified by lab-based methods may be downgraded to intermediate-risk when assessed using non-lab-based models, potentially leading to suboptimal clinical management.

These findings highlight the need to incorporate diabetes status into CVD prediction models to prevent risk misclassification. For example, the Framingham risk score, which includes self-reported diabetes demonstrated substantial agreement among participants diagnosed with diabetes [16]. While integrating self-reported diabetes may enhance predictive accuracy (overall as well as for participants with diabetes), further prospective validation using cardiovascular event data is essential. Clinicians should exercise caution when applying non-lab-based WHO-CVD score and GLOBORISK CVD-score in populations with diabetes to enhance their accuracy and clinical utility and avoid risk underestimation. Given the varying levels of agreement across different risk factor profiles, rigorous validation using prospective cohort study is crucial before incorporating non-lab-based models into healthcare systems to minimize the risk of CVD mismanagement.

The study has several notable strengths. First, the large sample size provided sufficient statistical power to calculate CVD risk scores using multiple models and to categorize participants into different risk groups (low, intermediate, and high). Second, the study included a comprehensive range of clinical assessments spanning anthropometric, biochemical, and cardiovascular parameters conducted using rigorous protocols to ensure high data quality. The use of standardized methods, such as the GOD-PAP assay for glucose and the Omron M5-I device for blood pressure measurement, enhances the reliability of the findings and facilitates comparability with other studies. Third, the study prioritized reproducibility, reporting high intraclass correlation coefficients for key variables, thereby reinforcing confidence in the reliability of the collected data. Fourth, the relationship between WHO- and GLOBORISK-CVD risk models and various measures of subclinical atherosclerosis ~~was~~were evaluated to explore the utility of these models in identifying individuals at high risk for CVD. While this approach is not the gold standard for validating CVD risk prediction models, it provides a degree of confidence in their applicability. Fifth, the analysis employed robust statistical methods, including quadratic weighted kappa statistics and Bland-Altman analysis, to assess agreement between models.

Limitations of the study include: Although the study is based on a large cohort, the rural, South Asian population might limit the generalizability of the findings to other populations or urban settings. Some important variables, such as smoking and alcohol use, are self-reported, which can introduce recall bias or social desirability bias. We excluded participants with missing values for relevant covariates from the analysis and we are not sure about this exclusion will introduce potential biases in the analysis.

Public health significance: The study’s findings underscore significant public health implications for India, where non-laboratory-based cardiovascular risk scores could greatly strengthen CVD prevention efforts. In many parts of India, access to laboratory tests is limited by cost and infrastructure, so a risk score that relies only on simple measures (age, blood pressure, BMI, smoking status) is particularly valuable. These non-lab risk scores can be integrated into national programs like the NPCDCS, which already deploys community health workers (ASHAs and ANMs) for population-based screening of hypertension and diabetes. Incorporating CVD risk scoring into such routine screening would enable frontline workers to identify high-risk individuals early and refer them for timely intervention. By facilitating cost-effective, community-level risk assessment and triage, the use of non-laboratory-based risk scores in India can contribute to large-scale CVD prevention and inform healthcare policy, ultimately helping to reduce the burden of cardiovascular diseases nationally.

Commented [KH9]: Should non-lab be used in diabetes population?

CONCLUSION

Using WHO and GLOBORISK CVD risk prediction models, we calculated 10-year CVD risk in an Indian population and observed high overall concordance between laboratory-based and non-laboratory-based assessments. However, the presence of systematic bias particularly in individuals with diabetes and elevated cholesterol levels underscores the need for prospective validation with cardiovascular event data before these models can be fully integrated into clinical practice in resource-limited settings.

Author Contributions: Conceptualisation, H.M.; Methodology, H.M., P.A.C.M., B.K. and S.K.; Software, H.M.; Validation, H.M., P.A.C.M., S.B., B.K. and S.K.; Formal analysis, H.M.; Investigation, S.B., B.K. and S.K.; Resources, B.K. and S.K.; Data curation, S.B.; Writing—original draft preparation, H.M.; Writing—review and editing, P.A.C.M., J.L., S.B., S.K.B., A.S., V.G., G.K.W, B.K. and S.K.; Supervision, S.K.; Project administration, B.K. and S.K.; Funding acquisition, S.K. All authors have read and agreed to the published version of the manuscript.

Funding Sources

This work was supported by The Wellcome Trust, UK (grant codes 083707, 084774 and 084754) and Medical Research Council, UK (grant codes MR/T038292/1 and MR/V001221/1) with in-kind support (infrastructure) from the Indian Council of Medical Research - National Institute of Nutrition, India.

Institutional Review Board Statement

The study was carried out in accordance with the principles of the Helsinki Declaration. The study protocol and tools have been approved by the ethics committees of ICMR-NIN (Application No. CR/1/V/2023; date: 21 October 2021) and Indian Institute of Public Health Hyderabad (IIPHH/TRCIEC/189/2018; date: 20 May 2020), India, and the London School of Hygiene and Tropical Medicine, UK [Application No. 21771/RR/19113; date: 18 March 2022].

Informed Consent Statement

We obtained verbal permission from the heads and governing committees of the villages. Written informed consent (or witnessed thumbprint if illiterate) for inclusion in the study was obtained from each participant prior to enrolment.

Data Availability Statement

The datasets analysed during the current study are available from the corresponding author on reasonable request.

Conflicts of Interest

The authors declare no conflicts of interest.

REFERENCES

1. Prabhakaran D, Jeemon P, Roy A: **Cardiovascular Diseases in India: Current Epidemiology and Future Directions**. *Circulation* 2016, **133**(16):1605-1620.
2. Prabhakaran D, Singh K, Roth GA, Banerjee A, Pagidipati NJ, Huffman MD: **Cardiovascular Diseases in India Compared With the United States**. *Journal of the American College of Cardiology* 2018, **72**(1):79-95.
3. Kalra A, Jose AP, Prabhakaran P, Kumar A, Agrawal A, Roy A, Bhargava B, Tandon N, Prabhakaran D: **The burgeoning cardiovascular disease epidemic in Indians - perspectives on contextual factors and potential solutions**. *The Lancet regional health Southeast Asia* 2023, **12**:100156.
4. McGorrian C, Yusuf S, Islam S, Jung H, Rangarajan S, Avezum A, Prabhakaran D, Almahmeed W, Rumboldt Z, Budaj A *et al*: **Estimating modifiable coronary heart disease risk in multiple regions of the world: the INTERHEART Modifiable Risk Score**. *European Heart Journal* 2011, **32**(5):581-589.
5. Kaptoge S, Pennells L, De Bacquer D, Cooney MT, Kavousi M, Stevens G, Riley LM, Savin S, Khan T, Altay S *et al*: **World Health Organization cardiovascular disease risk charts: revised models to estimate risk in 21 global regions**. *The Lancet Global Health* 2019, **7**(10):e1332-e1345.
6. D'Agostino RB, Sr., Vasan RS, Pencina MJ, Wolf PA, Cobain M, Massaro JM, Kannel WB: **General cardiovascular risk profile for use in primary care: the Framingham Heart Study**. *Circulation* 2008, **117**(6):743-753.
7. Visseren FLJ, Mach F, Smulders YM, Carballo D, Koskinas KC, Bäck M, Benetos A, Biffi A, Boavida J-M, Capodanno D *et al*: **2021 ESC Guidelines on cardiovascular disease prevention in clinical practice: Developed by the Task Force for cardiovascular disease prevention in clinical practice with representatives of the European Society of Cardiology and 12 medical societies With the special contribution of the European Association of Preventive Cardiology (EAPC)**. *European Heart Journal* 2021, **42**(34):3227-3337.
8. Badawy MAEMD, Naing L, Johar S, Ong S, Rahman HA, Tengah DSNAP, Chong CL, Tuah NAA: **Evaluation of cardiovascular diseases risk calculators for CVDs prevention and management: scoping review**. *BMC Public Health* 2022, **22**(1):1742.
9. Arnett DK, Blumenthal RS, Albert MA, Buroker AB, Goldberger ZD, Hahn EJ, Himmelfarb CD, Khera A, Lloyd-Jones D, McEvoy JW *et al*: **2019 ACC/AHA Guideline on the Primary Prevention of Cardiovascular Disease: A Report of the American College of Cardiology/American Heart Association Task Force on Clinical Practice Guidelines**. 2019, **140**(11):e596-e646.
10. Ueda P, Woodward M, Lu Y, Hajifathalian K, Al-Wotayan R, Aguilar-Salinas CA, Ahmadvand A, Azizi F, Bentham J, Cifkova R *et al*: **Laboratory-based and office-based risk scores and charts to predict 10-year risk of cardiovascular disease in 182 countries: a pooled analysis of prospective cohorts and health surveys**. *The lancet Diabetes & endocrinology* 2017, **5**(3):196-213.
11. **Directorate General of Health Services, Ministry of Health and Family Welfare, Government of India. Comprehensive primary health care NCD solution application ANM user manual. DGHS [Internet]. 2020 [cited 2024 Dec 15].**
12. **Ministry of Health and Family Welfare, Government of India. Nationwide program for NCD control and prevention launched. Press Information Bureau [Internet]. 2018 Aug 21 [cited 2024 Dec 15].**
13. **The Hans India. Hyderabad health survey inches to finish line. The Hans India [Internet]. 2021 Dec 15 [cited 2024 Dec 15].**
14. Ananda Selva Das P, Dubey M, Kaur R, Salve HR, Varghese C, Nongkynrih B: **WHO Non-Lab-Based CVD Risk Assessment: A Reliable Measure in a North Indian Population**. *Global Heart* 2022.
15. Sivanantham P, Kar SS, Lakshminarayanan S, Sahoo JP, Bobby Z, Varghese C: **Performance of WHO updated cardiovascular disease risk prediction charts in a low-resource setting - Findings from a community-based survey in Puducherry, India**. *Nutrition, metabolism, and cardiovascular diseases : NMCD* 2022, **32**(9):2129-2136.

16. Birhanu MM, Zengin A, Evans RG, Kim J, Olaiya MT, Riddell MA, Kalyanram K, Kartik K, Suresh O, Thomas N *et al*: **Comparison of laboratory-based and non-laboratory-based cardiovascular risk prediction tools in rural India**. *Tropical medicine & international health : TM & IH* 2025, **30**(1):57-64.
17. Joseph P, Yusuf S, Lee SF, Ibrahim Q, Teo K, Rangarajan S, Gupta R, Rosengren A, Lear SA, Avezum A *et al*: **Prognostic validation of a non-laboratory and a laboratory based cardiovascular disease risk score in multiple regions of the world**. *Heart* 2018, **104**(7):581.
18. Kinra S, Radha Krishna KV, Kuper H, Rameshwar Sarma KV, Prabhakaran P, Gupta V, Walia GK, Bhogadi S, Kulkarni B, Kumar A *et al*: **Cohort profile: Andhra Pradesh Children and Parents Study (APCAPS)**. *International journal of epidemiology* 2014, **43**(5):1417-1424.
19. Mahajan H, Lieber J, Carson Mallinson PA, Bhogadi S, Banjara SK, Kinra S, Kulkarni B: **The higher dietary inflammation is associated with a higher burden of multimorbidity of cardio-metabolic and mental health disorders in an urbanizing community of southern India: A cross-sectional analysis for the APCAPS cohort**. *Human nutrition & metabolism* 2024, **36**:200254.
20. Kasim SS, Ibrahim N, Malek S, Ibrahim KS, Aziz MF, Song C, Chia YC, Ramli AS, Negishi K, Mat Nasir N: **Validation of the general Framingham Risk Score (FRS), SCORE2, revised PCE and WHO CVD risk scores in an Asian population**. *The Lancet regional health Western Pacific* 2023, **35**:100742.
21. Mahajan H, Mallinson PAC, Lieber J, Bhogadi S, Banjara SK, Reddy VS, Reddy GB, Kulkarni B, Kinra S: **The Association of Total Meat Intake with Cardio-Metabolic Disease Risk Factors and Measures of Sub-Clinical Atherosclerosis in an Urbanising Community of Southern India: A Cross-Sectional Analysis for the APCAPS Cohort**. 2024, **16**(5):746.
22. Cohen J: **Weighted kappa: nominal scale agreement with provision for scaled disagreement or partial credit**. *Psychological bulletin* 1968, **70**(4):213-220.
23. Giavarina D: **Understanding Bland Altman analysis**. *Biochemia medica* 2015, **25**(2):141-151.
24. Landis JR, Koch GG: **The measurement of observer agreement for categorical data**. *Biometrics* 1977, **33**(1):159-174.
25. Bendera A, Nakamura K, Seino K, Alemi S: **Performance of the non-laboratory based 2019 WHO cardiovascular disease risk prediction chart in Eastern Sub-Saharan Africa**. *Nutrition, metabolism, and cardiovascular diseases : NMCD* 2024, **34**(6):1448-1455.
26. Dehghan A, Rayatinejad A, Khezri R, Aune D, Rezaei F: **Laboratory-based versus non-laboratory-based World Health Organization risk equations for assessment of cardiovascular disease risk**. *BMC medical research methodology* 2023, **23**(1):141.
27. Rezaei F, Seif M, Gandomkar A, Fattahi MR, Malekzadeh F, Sepanlou SG, Hasanzadeh J: **Comparison of laboratory-based and non-laboratory-based WHO cardiovascular disease risk charts: a population-based study**. *Journal of Translational Medicine* 2022, **20**(1):133.
28. Chhezom K, Gurung MS, Wangdi K: **Comparison of Laboratory and Non-Laboratory-Based 2019 World Health Organization Cardiovascular Risk Charts in the Bhutanese Population**. *Asia-Pacific journal of public health* 2024, **36**(1):29-35.

Figure Legends

Figure 1.0 Description of the study population and each risk score assessment groups

n, frequency; M, Male; F, Female; SBP, systolic blood pressure; HDL-C, high density lipoprotein-cholesterol; WHO-CVD, World Health Organization cardiovascular disease risk charts; CVD, cardiovascular disease; DM, type 2 diabetes mellitus; BMI, body mass index; MI, myocardial infarction; CHD, coronary heart disease; PAD, peripheral arterial disease;

TABLES

Table 1 Characteristics of APCAPS participants (follow-up 3) considered for the WHO CVD-risk prediction score (n= 2465)

Variables	Male (n=1184)	Female (n= 1281)
Age in years, mean (SD)	53.47 (6.73)	47.22 (5.47)
Smoking, n (%)	710 (60.0)	314 (24.5)
BMI in kg/m^2, mean (SD)	20.50 (3.67)	21.87 (4.14)
HDL-C in mg/dL, mean (SD)	45.2 (15.1)	44.8 (13.1)
Total Cholesterol mg/dL, mean (SD)	141.1 (87.4)	179.8 (37.5)
Systolic Blood Pressure in mmHg, mean (SD)	128.69 (19.34)	122.29 (16.15)
Antihypertensive medications, n (%)	87 (7.4)	70(5.5)
Fasting glucose in mg/dL, mean (SD)	99.3 (27.9)	98.0 (26.7)
Diabetes mellitus, n (%)	88 (7.4)	75 (5.9)
WHO Lab-based predicted CVD risk, median (IQR)	***6.0 (4.0, 9.0)***	***3 (2.0, 4.0)***
- Low, n (%)	892 (75.3)	1240 (96.8)
- Intermediate, n (%)	252 (21.3)	39 (3.0)
- High, n (%)	40 (3.4)	2 (0.2)
WHO non-Lab-based predicted CVD risk, median (IQR)	***6.0 (4.0, 9.0)***	***3 (2.0, 4.0)***
- Low, n (%)	913 (77.1)	1241 (96.9)
- Intermediate, n (%)	250 (21.1)	39 (3.0)
- High, n (%)	21 (1.8)	1 (0.1)
GLOBORISK Lab-based predicted CVD risk, median (IQR)	***12.0 (9.0, 16.0)***	***5.0 (3.0, 9.0)***
- Low, n (%)	353 (29.8)	1018 (79.5)
- Intermediate, n (%)	661 (55.8)	220 (17.2)
- High, n (%)	170 (14.4)	43 (3.4)
GLOBORISK non-Lab-based predicted CVD risk, median (IQR)	***15.0 (10.0, 16.0)***	***5.0 (3.0, 9.0)***
- Low, n (%)	233 (19.7)	973 (76.0)
- Intermediate, n (%)	788 (66.6)	253 (19.8)
- High, n (%)	163 (13.8)	55 (4.3)
Subclinical-atherosclerosis biomarkers		
- CIMT in mm, mean (SD)	1.001 (0.215)	0.985 (0.182)
- PWV in m/s, mean (SD)	8.02 (1.34)	7.64 (1.24)
- Augmentation Index (%), mean (SD)	29.44 (8.38)	30.40 (9.11)

%, Percentage; APCAPS, Andhra Pradesh Children and Parents Study; SD, standard deviation; IQR, interquartile range; CIMT, carotid intima media thickness; PWV, pulse wave velocity; and AIx, Augmentation Index; SD, standard deviation; WHO, world health organization; CVD, cardiovascular diseases; mm, millimeter; m/s, meter per second; HDL-C, high density lipoprotein cholesterol;

For CIMT, PWV, and AIx, data was available for 723, 715, and 697 for males and 836, 843, and 813 for females, respectively;

Table 2 Levels of CIMT, PWV and AIx across the categories of WHO-CVD and GLOBORISK scores as well as β coeff. (95% CI) for CIMT, PWV and AIx per SD change in CVD score

	CVD Risk Categories			p-trend	β Coeff. (95% CI) per SD change in CVD score
	**Low**	**Intermediate**	**High**		
**Carotid Intima Media Thickness (in mm)**					
Males					
WHO Lab-based CVD-Score	0.975 (0.957, 0.992)	1.076 (1.043, 1.110)	1.118 (1.031, 1204)	<0.001	0.048 (0.032, 0.064)
WHO non-Lab-based CVD-Score	0.977 (0.960, 0.995)	1.079 (1.045, 1.113)	1.127 (1.008, 1.247)	<0.001	0.048 (0.032, 0.064)
GLOBORISK Lab-based CVD- Score	0.940 (0.911, 0.969)	1.015 (0.994, 1.035)	1.061 (1.022, 1.100)	<0.001	0.041 (0.026, 0.057)
GLOBORISK non-Lab-based CVD- Score	0.929 (0.892, 0.966)	0.999 (0.981, 1.018)	1.098 (1.056, 1.140)	<0.001	0.041 (0.026, 0.056)
**Females**					
WHO Lab-based CVD-Score	0.983 (0.970, 0.995)	1.062 (0.986, 1.138)	1.11 (0.755, 1.466)	0.101	0.0307 (0.018, 0.044)
WHO non-Lab-based CVD-Score	0.984 (0.971, 0.996)	1.024 (0.948, 1.010)	1.11 (0.753, 1.467)	0.473	0.029 (0.017, 0.042)
GLOBORISK Lab-based CVD- Score	0.978 (0.964, 0.992)	1.011 (0.981, 1.042)	1.042 (0.963, 1.122)	0.051	0.029 (0.016, 0.042)
GLOBORISK non-Lab-based CVD- Score	0.970 (0.956, 0.984)	1.035 (1.008, 1.063)	1.016 (0.954, 1.078)	<0.001	0.026 (0.013, 0.038)
**Pulse Wave Velocity (in m/s)**					
	**CVD Risk Categories**			**p-trend**	**β Coeff. (95% CI) per SD change in CVD score**
	**Low**	**Intermediate**	**High**		
Males					
WHO Lab-based CVD-Score	7.71 (7.61, 7.82)	8.88 (8.69, 9.07)	9.35 (8.83, 9.88)	<0.001	0.579 (0.485, 0.672)
WHO non-Lab-based CVD-Score	7.74 (7.64, 7.84)	8.93 (8.73, 9.12)	10.10 (9.29, 10.90)	<0.001	0.603 (0.511, 0.696)
GLOBORISK Lab-based CVD- Score	7.64 (7.47, 7.82)	7.95 (7.82, 8.07)	8.94 (8.71, 9.18)	<0.001	0.421 (0.330, 0.513)
GLOBORISK non-Lab-based CVD- Score	7.55 (7.33, 7.77)	7.89 (7.78, 8.00)	9.27 (9.02, 9.51)	<0.001	0.504 (0.414, 0.593)
Females					
WHO Lab-based CVD-Score	7.61 (7.52, 7.69)	8.41 (7.89, 8.92)	9.37 (7.65, 11.08)	0.002	0.338 (0.255, 0.420)
WHO non-Lab-based CVD-Score	7.61 (7.52, 7.69)	8.54 (8.03, 9.05)	8.63 (6.21, 11.06)	0.001	0.314 (0.233, 0.396)
GLOBORISK Lab-based CVD- Score	7.53 (7.44, 7.63)	7.97 (7.77, 8.17)	8.58 (8.05, 9.10)	<0.001	0.286 (0.201, 0.370)
GLOBORISK non-Lab-based CVD- Score	7.57 (7.48, 7.67)	7.73 (7.54, 7.91)	8.30 (7.89, 8.71)	0.002	0.202 (0.119, 0.284)
**Augmentation Index (in %)**					
	**CVD Risk Categories**			**p-trend**	**β Coeff. (95% CI) per SD change in CVD score**
	**Low**	**Intermediate**	**High**		
Males					
WHO Lab-based CVD-Score	28.34 (27.65, 29.04)	33.16 (31.85, 34.46)	30.70 (27.03, 34.37)	<0.001	2.23 (1.58, 2.88)
WHO non-Lab-based CVD-Score	28.42 (27.73, 29.11)	33.07 (31.74, 34.40)	32.83 (27.17, 38.50)	<0.001	2.34 (1.71, 2.98)
GLOBORISK Lab-based CVD- Score	27.00 (25.86, 28.13)	29.46 (28.66, 30.26)	33.98 (32.42, 35.53)	<0.001	2.28 (1.65, 2.90)
GLOBORISK non-Lab-based CVD- Score	26.44 (24.98, 27.90)	29.24 (28.52, 29.96)	34.42 (32.75, 36.09)	<0.001	2.54 (1.93, 3.15)
Females					
WHO Lab-based CVD-Score	30.27 (29.63, 30.91)	34.38 (30.58, 38.18)	38.83 (26.22, 51.45)	0.050	1.98 (1.36, 2.60)
WHO non-Lab-based CVD-Score	30.25 (29.62, 30.89)	35.84 (31.95, 39.73)	35.33 (17.51, 53.15)	0.506	2.12 (1.51, 2.73)
GLOBORISK Lab-based CVD- Score	29.64 (28.95, 30.33)	33.43 (31.93, 34.94)	34.35 (30.40, 38.30)	<0.001	1.80 (1.16, 2.43)
GLOBORISK non-Lab-based CVD- Score	29.69 (28.98, 30.40)	32.30 (30.90, 33.70)	34.56 (31.47, 37.64)	<0.001	1.81 (1.19, 2.42)

β coeff, β coefficient; CI, confidence interval; mm, millimeter; m/s, meter per second; %, Percentage; CIMT, carotid intima media thickness; PWV, pulse wave velocity; and AIx, Augmentation Index; SD, standard deviation; WHO, world health organization; CVD, cardiovascular diseases;

Levels of CIMT, PWV, and AIx are expressed as mean (95% CI) across the categories of CVD-scores (low, intermediate, and high);

- For *males* One SD of WHO Lab-based CVD-Score, WHO non-Lab-based CVD-Score, GLOBORISK Lab-based CVD-Score, GLOBORISK non-Lab-based CVD-Score equal to 4.77, 4.29, 7.63, and 5.97, respectively;
- For *females* One SD of WHO Lab-based CVD-Score, WHO non-Lab-based CVD-Score, GLOBORISK Lab-based CVD-Score, GLOBORISK non-Lab-based CVD-Score equal to 2.54, 2.61, 5.75, and 5.46, respectively;

Table 3 Agreement between the lab- and non-lab-based WHO- and GLOBORISK-CVD-risk prediction scores

		**WHO CVD Risk Scores**		**GLOBORISK CVD Risk Scores**	
		**Percent Agreement (95% CI)**	**Quadratic Weighted Kappa (95% CI)**	**Percent Agreement (95% CI)**	**Quadratic Weighted Kappa (95% CI)**
Overall (n= 2465)		98.6 (98.4 – 98.9)	0.82 (0.78 – 0.85)	94.0 (93.5 – 94.5)	0.72 (0.69 – 0.74)
Sex	Men (n= 1184)	97.6 (97.2 – 98.1)	0.81 (0.77 – 0.85)	92.9 (92.2 – 93.6)	0.63 (0.59 – 0.67)
	Women (n= 1281)	99.5 (99.4 – 99.7)	0.73 (0.62 – 0.84)	95.0 (94.3 – 95.6)	0.63 (0.58 – 0.68)
Smoker	Yes (n= 1024)	97.4 (96.9 – 98.0)	0.81 (0.77 – 0.85)	92.0 (91.3 – 92.7)	0.48 (0.43 – 0.52)
	No (n= 1441)	99.5 (99.3 – 99.7)	0.66 (0.56 – 0.77)	95.4 (94.8 – 96.1)	0.55 (0.49 – 0.61)
Diabetes	Yes (n= 184)	91.0 (88.7 – 93.4)	0.53 (0.42 – 0.64)	78.0 (74.6 – 81.4)	0.42 (0.35 – 0.49)
	No (n= 2281)	99.2 (99.1 – 99.4)	0.88 (0.85 – 0.91)	95.3 (94.9 – 95.7)	0.76 (0.74 – 0.78)
SBP	≥140 mmHg (n= 472)	96.5 (95.6 – 97.4)	0.80 (0.75 – 0.85)	90.5 (89.2 – 91.8)	0.68 (0.63 – 0.73)
	<140 mmHg (n= 1993)	99.1 (98.9 – 99.4)	0.77 (0.71 – 0.83)	94.8 (94.3 – 95.3)	0.65 (0.62 – 0.68)
Total cholesterol	≥200 mg/dL (n= 608)	97.3 (96.5 – 98.1)	0.76 (0.67 – 0.82)	93.6 (92.4 – 94.9)	0.75 (0.70 – 0.80)
	<200 mg/dL (n= 1857)	99.0 (98.8 – 99.3)	0.85 (0.81 – 0.88)	94.1 (93.6 – 94.6)	0.70 (0.68 – 0.73)
BMI	≥25 kg/m^2 (n= 399)	97.7 (96.9 – 98.6)	0.74 (0.65 – 0.83)	92.2 (90.8 – 93.7)	0.69 (0.63 – 0.75)
	<25 kg/ m^2 (n= 2066)	98.8 (98.5 – 99.1)	0.83 (0.80 – 0.87)	94.3 (93.8 – 94.8)	0.72 (0.70 – 0.75)

CI, confidence interval; SBP, systolic blood pressure; BMI, body mass index;

Table 4 Mean difference and limits of agreement for the Bland-Altman analysis

CVD-prediction scores	Mean Difference (95% CI)	Limit of agreement		n (%) observations out of LOA
		Lower (95% CI)	Upper (95% CI)	
**WHO- CVD-Risk Prediction Score**				
Overall (n= 2465)	0.10 (0.03, 0.17)	-3.19 (-3.31, -3.08)	3.39 (3.28, 3.51)	111 (4.50)
Male (n= 1184)	0.31 (0.19, 0.43)	-3.87 (-4.08, -3.66)	4.49 (4.28, 4.70)	71 (6.00)
Female (n= 1281)	-0.09 (-0.15, -0.03)	-2.19 (-2.29, -2.09)	2.01 (1.91, 2.11)	42 (3.28)
Smokers (n= 1024)	0.04 (-0.09, 0.17)	-4.19 (-4.42, -3.96)	4.27 (4.04, 4.50)	54 (5.27)
Diabetes (n= 184)	4.17 (3.76, 4.58)	-1.34 (-2.04, -0.63)	9.68 (8.97, 10.38)	12 (6.52)
SBP ≥140 mmHg (n= 472)	0.09 (-0.15, 0.33)	-5.16 (-5.58, -4.74)	5.34 (4.92, 5.76)	25 (5.30)
Total Cholesterol ≥200 mg/dL (n= 608)	1.16 (0.98, 1.34)	-3.37 (-3.69, -3.05)	5.69 (5.37, 6.01)	35 (5.76)
BMI ≥25 kg/m^2 (n= 399)	-0.04 (-0.26, 0.18)	-4.37 (-4.75, -4.00)	4.29 (3.92, 4.67)	20 (5.01)
**GLOBORISK- CVD-Risk Prediction Score**				
Overall (n= 2465)	-0.53 (-0.70, -0.36)	-9.15 (-9.46, -8.85)	8.09 (7.79,8.40)	103 (4.18)
Male (n= 1184)	-0.66 (-0.95, -0.37)	-10.62 (-11.12, -10.11)	9.30 (8.80, 9.80)	52 (4.39)
Female (n= 1281)	-0.41 (-0.61, -0.21)	-7.56 (-7.91, -7.22)	6.74 (6.40, 7.09)	57 (4.45)
Smokers (n= 1024)	-1.15 (-1.48, -0.82)	-11.79 (-12.37, -11.22)	9.50 (8.92, 10.07)	48 (4.69)
Diabetes (n= 184)	10.44 (9.38, 11.50)	-3.89 (-5.72, -2.06)	24.77 (22.94, 26.60)	10 (5.43)
SBP ≥140 mmHg (n= 472)	-0.21 (-0.82, 0.40)	-13.40 (-14.45, -12.35)	12.98 (11.93, 14.03)	27 (5.72)
Total Cholesterol ≥200 mg/dL (n= 608)	2.85 (2.38, 3.32)	-8.85 (-9.67, -8.03)	14.55 (13.73, 15.37)	37 (6.09)
BMI ≥25 kg/m^2 (n= 399)	-0.005 (-0.59, 0.58)	-11.63 (-12.64, -10.62)	11.62 (10.61, 12.63)	23 (5.76)

n, frequency; %, percentage; CI, confidence interval; SBP, systolic blood pressure; BMI, body mass index;

Figure

Click here to access/download;Figure;CVD_WHO_GLOBORISK_Figure2.do

Figure 1. Description of the study population and each risk score assessment groups

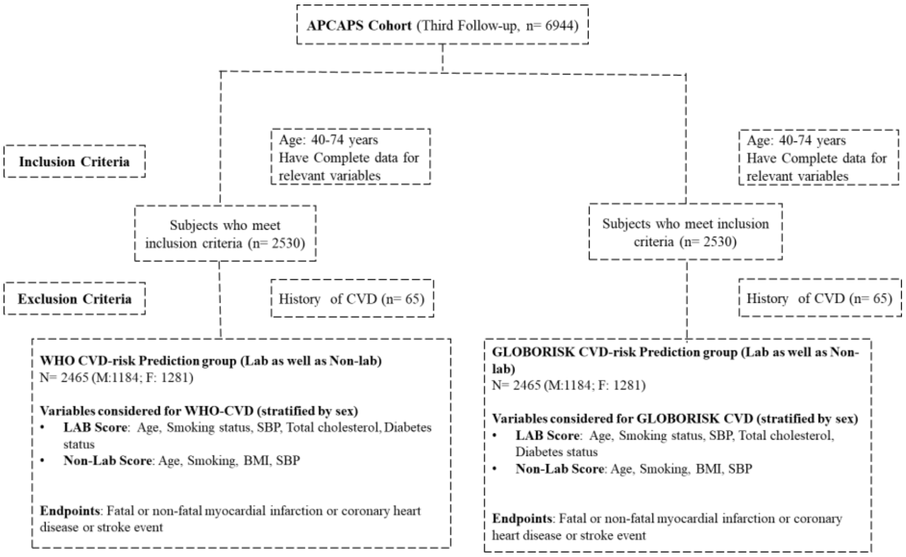

Supporting Information

Click here to access/download
Supporting Information
CVD_SUPPLEMENTARY_Material_PLOS_ONE.docx
