# Supplementary material for: Comparison of laboratory-based and non-laboratory-based WHO and GLOBORISK CVD risk scores: A cross-sectional analysis of the APCAPS cohort
Source: PLoS One. 2026 Feb 6;21(2):e0342471. doi: 10.1371/journal.pone.0342471 (PMC12880651; doi:10.1371/journal.pone.0342471)
Supplement: Attachment — Submitted filename: Review.docx [file pone.0342471.s009.docx]

**Estimating the Incidence of Heart Failure**

**Manuscript Number: PONE-D-25-32907**

Comments

The manuscript presents the results of a study aimed at comparing laboratory- and non-laboratory-based WHO and GLOBORISK cardiovascular risk scores, assess their concordance, and examine relationships with sub-clinical atherosclerosis in a rural Indian cohort including 2465 adults aged 47-74 years. The study analysed a cross-section of the cohort and used appropriate, established, statistical methods to assess categorical and continuous agreement and the linear association between the 4 risk scores and various sub-clinical atherosclerosis markers.

The introductory section clearly describes the rationale, aims and objectives of the study and its potential usefulness in a public health perspective. The methods section describes with sufficient detail the data collection and measurement procedures and the analytical approach. The results are clearly presented and contextualised in the Discussion section, and the conclusion are supported by the presented results. Major strengths and limitations of the study are discussed.

I have listed below some specific comments that I hope the Authors would consider addressing.

Major comments

1) Discussion

While I understand the focus of the study is the comparison between laboratory and non-laboratory risk scores (rather than the comparison between risk scores), I am still surprised by the absence of comments regarding the large discrepancies between the risk estimates between WHO and Globorisk (either lab or non-lab).

I wonder if it is possible to set aside some consideration regarding these differences when discussing – as the Authors do – the public health implications of their finding, by stating “*Consistent with previous research, our results support the broader* *applicability of these models, particularly in settings with limited laboratory resources or access*.” After deciding that non-lab scores are a good substitute of non-laboratory scores (with some exception that the Authors have clearly highlighted), can we ignore the problem of choosing which score is appropriate, considering that, in this population, median risk prediction are more than double in the Globorisk compared to the WHO score (even worse for the categorical distribution)? The Public Health Implications are difficult to ignore. Some of the highlighted differences between lab- and non-lab are almost negligible in relation to the between-models difference.

While this study is not aimed at comparing risk scores between them, I suggest that the discussion includes some considerations on this regard.

2) Estimation of Standard errors and confidence intervals

Reference [18] describes the original set-up of the cohort and indicated a sampling design which includes (if I interpret correctly the description) selection of villages on some specific areas, where then households and respondents are sampled. This multistage sampling strategy is known to influence the estimation of standard errors (usually inflating them). There is no mention in the analytical methods of any adjustement of the analyses at this regard.

I suggest the author consider the subject, either by including adjustment when conducting the statistical procedures or speculating on the potential consequences of not including one.

Also, please, specify the criteria of choosing 0.01 (as opposed to other cutoff) to take into account multiple testing.

Minor Comments

3) Page 12, line 216

“*Categorical variables were expressed as numbers (percentages), and continuous variables were presented as means and standard deviations (SDs)*”.

This sentence partially contradicts the following Results section where, for example, continuous CVD scores are reported (correctly, given their unlikely normal distribution) as median + IQR. Please, adapt the statement in the Methods section.

4) Page 12, line 220

“*To examine trends across CVD risk categories, median values were assigned to each category and treated as a continuous variable*”.

This sentence is not clear to me. To which categories does it refers? The three categories low, intermediate and high? I cannot find the specific analyses where this procedure has been applied.

5) Page 13, line 264

“…*plinear-trend <0.05*”.

Please, specify the use of a cut-off of 5% in relation to the statement in the Methods section that “*All p-values were two-tailed and a p-value less than 0.01 was considered significant …*”.

6) Title of Table 1

“*Characteristics of APCAPS participants (follow-up 3) considered for* ***the WHO CVD-risk prediction score*** *(n=2465)*”

The same sample is sued for both risk scores. Please, update the title to include both scores.

7) Data availability statements

The statement “*Yes - all data are fully available without restriction*” reported in the “Data Availability” section is in contradiction with the statement “*Data cannot be shared publicly because of Privacy policy of the Indian Council of Medical Research, India. Data are available from the ICMR-NIN Institutional Data Access / Ethics Committee (contact via email) for researchers who meet the criteria for access to confidential data*.” In the following section.

The statement reported in the Data Availability Statement section of the manuscript (“*The datasets analysed during the current study are available from the corresponding author on reasonable request*”) seems also contradicting the statement that the request should be address to the ICMR-NIN Institutional Data Access / Ethics Committee. Please, clarify and harmonise.
