# Supplementary material for: Comparison of laboratory-based and non-laboratory-based WHO and GLOBORISK CVD risk scores: A cross-sectional analysis of the APCAPS cohort
Source: PLoS One. 2026 Feb 6;21(2):e0342471. doi: 10.1371/journal.pone.0342471 (PMC12880651; doi:10.1371/journal.pone.0342471)
Supplement: Attachment — Submitted filename: 1_Response_to_reviewers.docx [file pone.0342471.s011.docx]

***Title of the manuscript:* Comparison of Laboratory-Based and Non-Laboratory-Based Who and GLOBORISK CVD Risk Scores: a Cross-Sectional Analysis of the APCAPS Cohort**

***Journal Requirements:***

When submitting your revision, we need you to address these additional requirements.

1. Please ensure that your manuscript meets PLOS ONE's style requirements, including those for file naming. The PLOS ONE style templates can be found at

<https://journals.plos.org/plosone/s/file?id=wjVg/PLOSOne_formatting_sample_main_body.pdf> and

[https://journals.plos.org/plosone/s/file?id=ba62/PLOSOne_formatting_sample_title_authors_affiliations.pdf](https://track.editorialmanager.com/CL0/https:%2F%2Fjournals.plos.org%2Fplosone%2Fs%2Ffile%3Fid=ba62%2FPLOSOne_formatting_sample_title_authors_affiliations.pdf/1/010f019add1386dd-fc309712-9fdb-4ad0-b936-ca3c00c77be0-000000/MVFYuw8naNxTm9XXXd1QphVvu_NixRxwxd3JZQOaaR4=239)

**RESPONSE**: Document formatted in PLOS ONE's style requirements.

2. We note that the grant information you provided in the ‘Funding Information’ and ‘Financial Disclosure’ sections do not match.

When you resubmit, please ensure that you provide the correct grant numbers for the awards you received for your study in the ‘Funding Information’ section.

**RESPONSE**: I have corrected the grant numbers. Now the grant information provided in the ‘Funding Information’ and ‘Financial Disclosure’ sections match.

“This work was supported by The Wellcome Trust, UK (Award Number: 083707, 084774 and 084754) and Medical Research Council, UK (Award Number: MR/T038292/1 and MR/V001221/1) with in-kind support (infrastructure) from the Indian Council of Medical Research - National Institute of Nutrition, India. The funders had no role in study design, data collection and analysis, decision to publish, or preparation of the manuscript”

3. Thank you for stating the following financial disclosure:
“This work was supported by The Wellcome Trust, UK (grant codes 083707, 084774 and 084754) and Medical Research Council, UK (grant codes MR/T038292/1 and MR/V001221/1) with in-kind support (infrastructure) from the Indian Council of Medical Research - National Institute of Nutrition, India.”

Please state what role the funders took in the study. If the funders had no role, please state: "The funders had no role in study design, data collection and analysis, decision to publish, or preparation of the manuscript."
If this statement is not correct you must amend it as needed.
Please include this amended Role of Funder statement in your cover letter; we will change the online submission form on your behalf.

**RESPONSE**: The statement saying “The funders had no role in study design, data collection and analysis, decision to publish, or preparation of the manuscript" has been added in the revised manuscripts.

4. We note that you have indicated that there are restrictions to data sharing for this study. For studies involving human research participant data or other sensitive data, we encourage authors to share de-identified or anonymized data. However, when data cannot be publicly shared for ethical reasons, we allow authors to make their data sets available upon request. For information on unacceptable data access restrictions, please see <http://journals.plos.org/plosone/s/data-availability#loc-unacceptable-data-access-restrictions>.

Before we proceed with your manuscript, please address the following prompts:

a) If there are ethical or legal restrictions on sharing a de-identified data set, please explain them in detail (e.g., data contain potentially identifying or sensitive patient information, data are owned by a third-party organization, etc.) and who has imposed them (e.g., a Research Ethics Committee or Institutional Review Board, etc.). Please also provide contact information for a data access committee, ethics committee, or other institutional body to which data requests may be sent.

**RESPONSE**: Ethical approval for the study was obtained from the following institutions:

- London School of Hygiene & Tropical Medicine, London, UK [LSHTM Ethics Ref: 21771]
- ICMR–National Institute of Nutrition (NIN), Hyderabad, India [NIN Protocol Number: 04/01/2020]
- Indian Institute of Public Health, Hyderabad, India [IIPHH/TRCIEC/189/2018]

The de-identified dataset cannot be shared publicly due to the privacy policy of the Indian Council of Medical Research (ICMR), which restricts open data sharing to protect participant confidentiality. These restrictions are in place to ensure compliance with national data protection regulations and ethical standards concerning sensitive health information.

Researchers who meet the criteria for accessing confidential data may request access through the Institutional Data Access/Ethics Committee of ICMR-NIN. Requests can be directed to:

**Dr. Shamanna BR**
Chair-person, Institutional Ethics Committee
Email: brsham@uohyd.ac.in
b) If there are no restrictions, please upload the minimal anonymized data set necessary to replicate your study findings to a stable, public repository and provide us with the relevant URLs, DOIs, or accession numbers. Please see <http://www.bmj.com/content/340/bmj.c181.long> for guidelines on how to de-identify and prepare clinical data for publication. For a list of recommended repositories, please see <https://journals.plos.org/plosone/s/recommended-repositories>. You also have the option of uploading the data as Supporting Information files, but we would recommend depositing data directly to a data repository if possible.
Please update your Data Availability statement in the submission form accordingly.

5. Please include captions for your Supporting Information files at the end of your manuscript, and update any in-text citations to match accordingly. Please see our Supporting Information guidelines for more information: <http://journals.plos.org/plosone/s/supporting-information>.

**RESPONSE**: Captions for Supporting Information files included at end of manuscript.

6. If the reviewer comments include a recommendation to cite specific previously published works, please review and evaluate these publications to determine whether they are relevant and should be cited. There is no requirement to cite these works unless the editor has indicated otherwise.

**RESPONSE**: Thank you for the information.

**Response to Reviewer Comments**

***Reviewer #2:***

1. **There should be comments on the relationship of CVD risk scores with subclinical atherosclerosis parameters measured.**

***RESPONSE*:** Thank you for this helpful suggestion. We have now expanded the Results and Discussion to more explicitly describe the associations between the WHO and GLOBORISK CVD risk scores and the subclinical atherosclerosis markers (CIMT, PWV and AIx). In the ‘Results’ section, we clarify that higher WHO and GLOBORISK risk categories were associated with progressively higher mean CIMT, PWV and AIx, and that each standard deviation increases in both laboratory- and non-laboratory-based scores was significantly associated with higher levels of all three markers in both men and women (Table 2). In the ‘Discussion’, we now emphasise that these positive, dose-response relationships between risk scores and subclinical atherosclerosis support the *construct validity* of both *WHO and GLOBORISK algorithms* in this rural South Asian cohort, and indicate that individuals classified as being at higher 10-year CVD risk have already accumulated structural and functional vascular changes.

2. **There should be comments on the disparity in CVD risk scores between men and women. This cannot be overlooked as the disparity is huge**

***Response***: We agree that the sex differences in predicted CVD risk are striking and have now made these patterns more explicit in both the ‘Results’ and ‘Discussion’. In our cohort, men were older on average (53.5 vs 47.2 years) and substantially more likely to be current smokers (60% vs 24.5%), with slightly higher systolic blood pressure than women. These differences in underlying risk-factor profiles, together with the sex-specific baseline hazards built into both the WHO and GLOBORISK algorithms, contribute to the higher absolute 10-year risk estimates in men.

Specifically, median WHO risk was 6% in men versus 3% in women, and 22-25% of men but only 3% of women had WHO-predicted risk ≥10%. For GLOBORISK, 70-80% of men but ~21-24% of women had a predicted 10-year risk ≥10%, despite similar or lower total cholesterol levels in men. These findings are consistent with the higher age-specific CVD incidence typically observed in men and with the design of the sex-specific charts.

Importantly, when we examined within-sex associations between risk scores and subclinical atherosclerosis markers (CIMT, PWV and AIx), we observed clear, *positive dose-response relationships in both men and women*, and similar increases in these markers per SD increase in WHO and GLOBORISK risk scores. This suggests that although absolute predicted risk is lower in women, the risk scores still discriminate vascular burden within women in a biologically plausible way. We now discuss these points explicitly in the revised ‘*Discussion*’ and highlight that absolute risk comparisons between sexes should be interpreted cautiously, while emphasizing the importance of sex-stratified application of these tools in clinical and public-health settings.

3. **There should also be a comment on WHO vs Globorisk as there are differences and what could have accounted for these differences**

***RESPONSE***: We agree with the reviewer that the differences between WHO and GLOBORISK estimates merit explicit comment. In our data, GLOBORISK consistently assigned higher 10-year CVD risk than the WHO charts, particularly among men. For example, while the median WHO 10-year risk in men was 6% (IQR 4-9%), only about 22-25% of men had WHO-predicted risk ≥10%; in contrast, the median GLOBORISK risk was 12-15%, and approximately 70-80% of men were classified as having ≥10% 10-year risk. Among women, WHO classified only ~3% as ≥10% risk, whereas GLOBORISK classified around 21-24% as ≥10% risk, despite similar median risks of 3% (WHO) and 5% (GLOBORISK).

These discrepancies largely reflect differences in how the *two tools are derived and calibrated*. In this analysis we used *WHO charts calibrated to the South Asia region*, which rely on regional age- and sex-specific incidence of myocardial infarction and stroke and average regional distributions of major risk factors. By contrast, we used the *India-calibrated GLOBORISK equations*, which are explicitly recalibrated to country-specific age- and sex-specific CVD mortality rates and national risk-factor distributions. In a setting such as India, where CVD mortality is higher than in several neighbouring countries, GLOBORISK therefore “anchors” the baseline hazard to a higher underlying national risk than the South-Asia-wide WHO charts, and consequently yields higher absolute 10-year risk estimates. Conceptually, both scores use very similar predictors (age, smoking, blood pressure, diabetes, total cholesterol or BMI), and both were developed for combined fatal and non-fatal coronary heart disease and stroke outcomes, suggesting that their relative ranking of individuals is driven by comparable risk-factor gradients, whereas the absolute level of predicted risk is more sensitive to the choice of calibration data.

Importantly, despite these differences in absolute levels and the proportion classified as “high risk”, both WHO and GLOBORISK scores showed very similar dose-response associations with subclinical atherosclerosis in our cohort: higher categories and each SD increase in either score were associated with higher CIMT, PWV and AIx in both sexes. This pattern suggests that *the two tools discriminate risk in a broadly comparable way but differ mainly in calibration of absolute risk*. We now highlight these points in the ‘Discussion’ and caution that, until prospective Indian event data allow formal calibration and head-to-head validation, programmatic decisions about which tool and threshold to adopt should consider that India-calibrated GLOBORISK will identify a substantially larger proportion of adults especially men as eligible for intensified preventive interventions than the South-Asia-calibrated WHO charts.

4. **What is your comment on *smoking status and its mean difference* on Bland Altman analysis**

***RESPONSE***: We thank the reviewer for raising the issue of smoking status in relation to the Bland-Altman analysis. In our stratified analyses, the agreement between laboratory-based and non-laboratory-based scores among smokers remained high for the WHO charts and showed only a modest systematic bias for the GLOBORISK charts. For WHO, the mean difference in smokers was 0.04 percentage points (95% CI: -0.09, 0.17), with limits of agreement from -4.19 to 4.27, indicating virtually no systematic bias between lab- and non-lab-based WHO scores among current smokers. In contrast, for GLOBORISK the mean difference in smokers was -1.15 percentage points (95% CI: -1.48, -0.82), meaning that non-laboratory-based GLOBORISK scores tend to classify smokers at slightly *higher* 10-year CVD risk than their laboratory-based counterparts, by just over one percentage point on average. Although this difference is statistically significant, its absolute magnitude is small compared with the much larger biases seen in participants with diabetes (mean difference 10.44%) or total cholesterol ≥200 mg/dL (2.85%), and the limits of agreement remain comparable to those observed in the overall sample. Consistent with these findings, categorical agreement among smokers is still substantial for WHO (percent agreement 97.4%, κ=0.81) and moderate for GLOBORISK (percent agreement 92.0%, κ=0.48). Taken together, these results suggest that smoking status does not materially compromise the concordance between lab- and non-lab-based WHO scores, and that for GLOBORISK the non-lab algorithm provides a slightly more conservative (higher) risk estimate in smokers rather than underestimating their risk.

**Minor Comments**

1. Is this still an on-going study. If no, then it should be “was”

**Response**: The study is ongoing.

2. Was LDL-C derived?

**Response**: Yes, using Friedewald’s Formula.

3. Did you consider peripheral artery disease?

**Response**: Both WHO and GLOBORISK CVD risk Score don’t consider peripheral artery disease.

Was this done (The validity of WHO-CVD and GLOBORISK risk prediction scores) as part of the earlier study in 2010-2012 or this was done by the current authors?

**Response**: The measurements CIMT, PWV, and Augmentation Index were measured in 2010-12 (APCPAS follow-up 3); The validity of the CVD risk scores against these measures were done in the current anlysis.

**REVIEWER 1 COMMENTS**:

The manuscript presents the results of a study aimed at comparing laboratory- and non-laboratory-based WHO and GLOBORISK cardiovascular risk scores, assess their concordance, and examine relationships with sub-clinical atherosclerosis in a rural Indian cohort including 2465 adults aged 47-74 years. The study analysed a cross-section of the cohort and used appropriate, established, statistical methods to assess categorical and continuous agreement and the linear association between the 4 risk scores and various sub-clinical atherosclerosis markers. The introductory section clearly describes the rationale, aims and objectives of the study and its potential usefulness in a public health perspective. The methods section describes with sufficient detail the data collection and measurement procedures and the analytical approach. The results are clearly presented and contextualised in the Discussion section, and the conclusion are supported by the presented results. Major strengths and limitations of the study are discussed. I have listed below some specific comments that I hope the Authors would consider addressing.

***Major comments***

**1) Discussion**

While I understand the focus of the study is the comparison between laboratory and non-laboratory risk scores (rather than the comparison between risk scores), I am still surprised by the absence of comments regarding the large discrepancies between the risk estimates between WHO and Globorisk (either lab or non-lab). I wonder if it is possible to set aside some consideration regarding these differences when discussing – as the Authors do – the public health implications of their finding, by stating “Consistent with previous research, our results support the broader applicability of these models, particularly in settings with limited laboratory resources or access.” After deciding that non-lab scores are a good substitute of non- laboratory scores (with some exception that the Authors have clearly highlighted), can we ignore the problem of choosing which score is appropriate, considering that, in this population, median risk prediction are more than double in the Globorisk compared to the WHO score (even worse for the categorical distribution)? The Public Health Implications are difficult to ignore. Some of the highlighted differences between lab- and non-lab are almost negligible in relation to the between-models difference. While this study is not aimed at comparing risk scores between them, I suggest that the discussion includes some considerations on this regard.

**RESPONSE**: We thank the reviewer for this very important point and agree that the discrepancies between WHO and GLOBORISK estimates have substantial public-health implications and deserve explicit discussion. Our original focus was on agreement between laboratory- and non-laboratory-based algorithms *within* each model, but as the reviewer notes, the *between-model* differences in absolute risk are much larger than the within-model differences and cannot be ignored.

In our cohort, WHO charts calibrated for South Asia yielded substantially lower 10-year CVD risk than the India-calibrated GLOBORISK equations, particularly in men. For example, among men the median WHO risk was 6% (IQR 4-9%) for both lab and non-lab models, whereas the corresponding GLOBORISK medians were 12-15%; 22-25% of men had WHO-predicted risk ≥10%, compared with 70-80% for GLOBORISK. Among women, WHO classified only about 3% as ≥10% risk, whereas GLOBORISK classified roughly 21-24% of women above this threshold, despite similar median risks of 3% vs 5%. These patterns underline that the choice of risk score will dramatically influence how many individuals are labelled “elevated risk” or become eligible for intensified prevention.

We now discuss several reasons that may account for these differences. First, although WHO and GLOBORISK use a similar set of predictors (age, sex, smoking, blood pressure, diabetes, total cholesterol or BMI), they are calibrated differently: in this analysis we used WHO charts calibrated to the broader South Asia region, whereas GLOBORISK is recalibrated to India-specific age- and sex-specific CVD mortality rates and national risk-factor distributions. In a context where CVD mortality in India is higher than in several neighbouring countries, the India-specific calibration used by GLOBORISK is expected to generate higher baseline hazards, and therefore higher absolute 10-year risk estimates, than region-level WHO charts. Second, small structural differences between the underlying equations (continuous risk functions versus charted categories) and the way age and risk factors enter the models may further accentuate these discrepancies at older ages and in higher-risk profiles.

At the same time, our analyses show that *relative* risk discrimination is broadly similar across the two tools. Within each sex, higher categories and each SD increase in either WHO or GLOBORISK scores were associated with higher CIMT, faster PWV and higher AIx, with consistent dose-response relationships for both laboratory- and non-laboratory-based algorithms. This suggests that both models rank individuals by underlying vascular burden in a comparable fashion, and that the main divergence lies in the calibration of absolute risk rather than in the ordering of individuals.

In response to the reviewer’s concern, we have revised the Discussion to (i) explicitly describe these between-model differences in median risk and risk-category distributions; (ii) explain how differences in regional versus country-specific calibration may underlie the higher GLOBORISK estimates; and (iii) temper our public-health interpretation. Specifically, we now state that although our findings support the use of *non-laboratory* algorithms as acceptable substitutes for their laboratory counterparts within a chosen model, the selection between WHO and GLOBORISK itself is not a neutral decision: in our setting, adopting India-calibrated GLOBORISK rather than WHO charts would result in a much larger proportion of adults especially men being classified above commonly used treatment thresholds. We also emphasise that, because our cross-sectional study does not include prospective CVD events, we cannot determine which calibration (WHO vs GLOBORISK) is more appropriate for India, and we call for head-to-head validation against hard outcomes before national guidelines choose one model and threshold for large-scale implementation.

We believe these additions directly address the reviewer’s concern and make clearer that our main conclusion that non-laboratory algorithms perform well relative to laboratory-based versions must be interpreted alongside the important policy choice of *which* risk equation to implement.

**2) Estimation of Standard errors and confidence intervals Reference [18] describes the original set-up of the cohort and indicated a sampling design which includes (if I interpret correctly the description) selection of villages on some specific areas, where then households and respondents are sampled. This multistage sampling strategy is known to influence the estimation of standard errors (usually inflating them). There is no mention in the analytical methods of any adjustement of the analyses at this regard. I suggest the author consider the subject, either by including adjustment when conducting the statistical procedures or speculating on the potential consequences of not including one.**

**Also, please, specify the criteria of choosing 0.01 (as opposed to other cutoff) to take into account multiple testing.**

***RESPONSE***: We thank the reviewer for raising this important point regarding the estimation of standard errors and confidence intervals. As described in the APCAPS cohort profile and in our Methods section, APCAPS is a long-term cohort based in 29 rural villages and small towns around Hyderabad, originally derived from a pregnancy nutrition trial and later expanded to include family members of the trial participants. Thus, participants are clustered within villages, but the cohort was not designed as a probability sample to generate population-representative weighted estimates. Our primary objective in the present analysis was to examine internal associations between different CVD risk scores and subclinical atherosclerosis markers within this cohort, rather than to produce nationally representative prevalence estimates. For this reason, the original analyses did not incorporate a formal survey design with sampling weights, and individuals were treated as independent observations.

We agree that village-level clustering can, in principle, lead to some underestimation of standard errors and overly narrow confidence intervals. In response to the reviewer’s concern, we have now (i) clarified the nature of the APCAPS sampling strategy in the Methods, and (ii) explicitly acknowledged in the Discussion that not formally accounting for clustering at the village level may have slightly underestimated standard errors. Importantly, the main associations between all four CVD risk scores and the three subclinical atherosclerosis measures are large in magnitude and highly statistically significant, with most p-values for trend <0.001 and narrow confidence intervals (Table 2), so any modest inflation of standard errors due to clustering is unlikely to change our substantive conclusions.

Regarding the choice of a significance threshold of p<0.01, our intention was to adopt a more conservative criterion a priori to partially account for multiple comparisons across three correlated subclinical atherosclerosis markers (CIMT, PWV and AIx) and four CVD risk scores. We did not apply a strict Bonferroni correction (which would yield a very low α, given the non-independence of these tests) because the outcomes and risk scores are moderately correlated, and our emphasis was on the size, direction and consistency of the associations across models rather than on isolated p-values. Instead, we prespecified p<0.01 as a pragmatic compromise to reduce the likelihood of false-positive findings while retaining adequate power. We have now clarified this rationale in the Statistical Methods section. In practice, nearly all key associations meet even more stringent thresholds (e.g. p<0.001), indicating that our main findings are robust to the specific cut-off chosen.

*Minor Comments*

3) Page 12, line 216

“Categorical variables were expressed as numbers (percentages), and continuous

variables were presented as means and standard deviations (SDs)”.

This sentence partially contradicts the following Results section where, for example,

continuous CVD scores are reported (correctly, given their unlikely normal

distribution) as median + IQR. Please, adapt the statement in the Methods section.

RESPONSE: Thank you for the comment. Appropriate corrections are made in the statistical analysis section and highlighted in yellow.

4) Page 12, line 220

“To examine trends across CVD risk categories, median values were assigned to each

category and treated as a continuous variable”.

This sentence is not clear to me. To which categories does it refers? The three

categories low, intermediate and high? I cannot find the specific analyses where this

procedure has been applied.

RESPONSE: Categories refer to low, intermediate and high-risk categories of CVD scores. CVD risk categorization is mentioned in the line page 6, 210-12

5) Page 13, line 264

“…plinear-trend &lt;0.05”.

Please, specify the use of a cut-off of 5% in relation to the statement in the Methods

section that “All p-values were two-tailed and a p-value less than 0.01 was

considered significant …”.

RESPONSE: Thank you for pointing out the mistake. I have changed the p-value for linear-trend to <0.01

**6) Title of Table 1 “Characteristics of APCAPS participants (follow-up 3) considered for the WHO CVD-risk prediction score (n=2465)” The same sample is sued for both risk scores. Please, update the title to include both scores.**

RESPONSE: Appropriate corrections are made in the heading of table 1 and highlighted in yellow.

**7) Data availability statements**

The statement “Yes - all data are fully available without restriction” reported in the “Data Availability” section is in contradiction with the statement “Data cannot be shared publicly because of Privacy policy of the Indian Council of Medical Research, India. Data are available from the ICMR-NIN Institutional Data Access / Ethics Committee (contact via email) for researchers who meet the criteria for access to confidential data.” In the following section.

The statement reported in the Data Availability Statement section of the manuscript (“The datasets analysed during the current study are available from the corresponding author on reasonable request”) seems also contradicting the statement that the request should be address to the ICMR-NIN Institutional Data Access / Ethics Committee. Please, clarify and harmonise.

RESPONSE: Appropriate corrections are made Data availability statements and harmonized it with Data availability Statement Section of the Manuscript.
